# Association of Short-Term Exposure to Ambient Fine Particulate Matter with Skin Symptoms in Schoolchildren: A Panel Study in a Rural Area of Western Japan

**DOI:** 10.3390/ijerph14030299

**Published:** 2017-03-13

**Authors:** Masanari Watanabe, Hisashi Noma, Jun Kurai, Hiroyuki Sano, Kyoko Iwata, Degejirihu Hantan, Yuji Tohda, Eiji Shimizu

**Affiliations:** 1 Department of Respiratory Medicine and Rheumatology, Faculty of Medicine, Tottori University, 36-1 Nishi-cho, Yonago 683-8504, Japan; junkurajun@gmail.com (J.K.); iwatak@mfc.or.jp (K.I.); degujirefu@med.tottori-u.ac.jp (D.H.); eiji@med.tottori-u.ac.jp (E.S.); 2 Department of Data Science, Institute of Statistical Mathematics, 10-3 Midori-cho, Tachikawa, Tokyo 190-8562, Japan; noma@ism.ac.jp; 3 Department of Respiratory Medicine and Allergology, Faculty of Medicine, Kinki University, 377-2 Ohnohigashi, Osakasayama 589-0014, Japan; hsano@med.kindai.ac.jp (H.S.); tohda@med.kindai.ac.jp (Y.T.); 4 Mio Fertility Clinic, Reproductive Centre, 2-1-1 Kuzumo-minami, Yonago 683-0008, Japan

**Keywords:** ambient particulate matter, Asian dust, particulate matter less than 2.5 μm, schoolchildren, skin symptoms

## Abstract

Numerous studies have unmasked the deleterious effects of particulate matter less than 2.5 μm (PM_2.5_) on health. However, epidemiologic evidence focusing on the effects of PM_2.5_ on skin health remains limited. An important aspect of Asian dust (AD) in relationship to health is the amount of PM_2.5_ contained therein. Several studies have demonstrated that AD can aggravate skin symptoms. The current study aimed to investigate the effects of short-term exposure to PM_2.5_ and AD particles on skin symptoms in schoolchildren. A total of 339 children recorded daily skin symptom scores during February 2015. Light detection and ranging were used to calculate AD particle size. Generalized estimating equation logistic regression analyses were used to estimate the associations among skin symptoms and the daily levels of PM_2.5_ and AD particles. Increases in the levels of PM_2.5_ and AD particles were not related to an increased risk of skin symptom events, with increases of 10.1 μg/m^3^ in PM_2.5_ and 0.01 km^−1^ in AD particles changing odds ratios by 1.03 and 0.99, respectively. These results suggest that short-term exposure to PM_2.5_ and AD does not impact skin symptoms in schoolchildren.

## 1. Introduction

Ambient particulate matter (PM) is a mixture of solid particles and liquid droplets originating from various natural and anthropogenic sources and comprises an important source of air pollutants [1]. Clinical, mechanistic, and epidemiological evidence have demonstrated adverse health effects from short-term and long-term exposure to ambient PM; these adverse influences are of concern to governments of various countries as well as the World Health Organization [2,3,4]. However, most epidemiological studies regarding the negative health effects of ambient PM have focused on respiratory and cardiovascular injuries.

The skin is the outermost barrier and is directly exposed to various environmental pollutants. Ultraviolet radiation from sunlight has been the most studied environmental hazard, and its consequences on skin are well established [5]. While a number of clinical and epidemiological studies highlight the associations with ambient PM and health effects, very little research is available to date concerning skin effects [5,6]. Furthermore, the exact mechanism of skin damage by ambient PM has yet to be elucidated. However, diesel-exhaust particles, which are an important source of PM less than 2.5 μm in diameter (PM_2.5_), have been shown to induce a strong inflammatory response in human skin cells [7,8]. Asian dust (AD), which originates in the deserts of East Asia, is the second strongest source of dust emissions worldwide (accounting for about 20% of the global total) and increases the concentrations of air pollutants including PM_2.5_ [9,10]. In addition, recent studies reported that AD was associated with an increase in mortality as well as emergency treatment for cardiovascular and respiratory diseases [11,12,13,14]. Moreover, Otani et al. found an adverse effect of AD on skin health in Japan [15]. These results suggest that short-term exposure to PM_2.5_ can aggravate skin symptoms.

Few studies have investigated the association between ambient PM and skin symptoms in children. Therefore, this study aimed to investigate the association between skin symptoms in children and short-term exposure to PM_2.5_ in western Japan. We also studied the relationship between skin symptoms in children and AD because AD is one of the important sources of PM_2.5_ in Japan.

## 2. Materials and Methods

### 2.1. Study Design

In this panel study, skin symptoms of schoolchildren were monitored daily in the morning during February 2015. The study was performed in Matsue, the capital city of Shimane Prefecture, in southwest Japan. This city houses approximately 200,000 individuals and covers an area of 530.2 km^2^. Four elementary schools were selected from a total of 35 in Matsue City because these four schools are located near principal roads in the central part of Matsue City and are within 10 km of each other as well as from the observatory for monitoring air pollution in Matsue City. All elementary schools agreed to participate in the study. All subjects lived within a 1-km radius of the schools. A total of 345 students aged 10 to 12 years in 2015 were enrolled. 

The study was approved by the institutional ethics committee (Ethics Committee of the Faculty of Medicine, Tottori University, approval number 2473). The study was also approved by the Matsue City Board of Education. The children and their parents were informed by teachers and provided written consent.

### 2.2. Recording of Daily Skin Symptoms

In January 2015, the schoolteachers provided information regarding the sex, height, and weight of each child based on physical measurements obtained by each school and recorded this information in a logbook for recording skin symptoms by teachers. The parents filled their children’s data concerning presence of asthma, allergic rhinitis, allergic conjunctivitis, atopic dermatitis, and food allergies in the logbook. The subjects were considered to have asthma if they met any of the following criteria in the past 12 months: diagnosis of asthma by a pediatrician, presence of wheezing, use of asthma medication, or a visit to a hospital for asthma. The subjects were considered to have allergic rhinitis, allergic conjunctivitis, atopic dermatitis, and/or food allergy if they met any of the following criteria in the past 12 months: diagnosis of any of these conditions by a pediatrician, use of medication for any of these conditions, or a visit to a hospital for any of these conditions. From 1 February 2015 to 28 February 2015, each child recorded daily scores for itchiness and rash as skin symptoms; scores were recorded as 0 (no symptoms), 1 (mild symptoms), or 2 (severe symptoms). Scores were recorded between 8:00 a.m. and 9:00 a.m. All children went to school on foot and were potentially exposed to any air pollutants.

### 2.3. Measurement of Air Pollutant Levels

Daily average concentrations of PM_2.5_, sulfur dioxide (SO_2_), nitrogen dioxide (NO_2_), and ozone in Matsue City were calculated based on data from the Japanese Ministry of the Environment, which monitors and reports hourly concentrations of these air pollutants. Meteorological variables, such as daily average levels of temperature, humidity, wind speed, and atmospheric pressure, were obtained from the Japan Meteorological Agency. The data were used to examine the associations between changes in skin symptoms and air pollutant levels.

Light Detection and Ranging (LIDAR) depolarization measurements performed simultaneously at two wavelengths can be used to identify non-spherical dust particles such as airborne sand dust particles and spherical particles such as air pollution aerosols in real time [16,17]. The LIDAR system can be used to measure the level of AD [16,17]; recently, a few studies from Japan used LIDAR data to estimate the effects of AD on health [18,19,20]. Daily particle levels were determined based on the median value of 96 measurements collected over a 24-h period from midnight of one day to midnight of the next day. The daily levels were only calculated when the number of available measurements exceeded 50% of the total number of measurements. This study used values measured from 120 m to 150 m above ground, which is the minimum altitude required by LIDAR systems to measure non-spherical and spherical particles. Data for AD concentrations from LIDAR were obtained from the Matsue observatory.

### 2.4. Statistical Analysis

To adequately address correlations among repeated measurements within a subject, generalized estimating equation (GEE) logistic regression analyses were used to estimate the associations among the daily skin symptoms of children and the daily average levels of PM_2.5_, SO_2_, NO_2_, and ozone and median levels of AD particles [21,22]. A skin symptom event was defined as a daily skin symptom score ≥2. The GEE logistic regression models included individual characteristics (sex, height, weight, asthma, allergic rhinitis, allergic conjunctivitis, atopic dermatitis, and food allergies) and meteorological variables (daily temperature, humidity, and atmospheric pressure) [23,24,25,26]. Estimates are given as the odds ratio in skin symptom events per interquartile range (IQR) change of PM_2.5_, SO_2_, NO_2_, ozone, and AD concentrations, with 95% confidence intervals (CIs). The working correlation matrices were set to exchangeable, and robust variance estimators were adopted for constructing the CIs for the odds ratios. Multiple imputations with chained equations were used for treating missing data; this adequately addresses the uncertainty of multiply generated prediction values for missing data [27]. The two-pollutant models were applied to different combinations of air pollutants (SO_2_, NO_2_, and ozone) to assess the stability of the effect of PM_2.5_ and AD on skin symptoms after adjustment for individual characteristics (age, sex, height, weight, and presence of asthma, allergic rhinitis, allergic conjunctivitis, atopic dermatitis, and food allergies), and meteorological variables (temperature, humidity, and atmospheric pressure). The GEE analyses were performed using R version 3.2.2 (R Foundation for Statistical Computing, Vienna, Austria).

## 3. Results

### 3.1. Subject Characteristics

Of the 345 children who were recruited, six were excluded because they failed to maintain a daily record of skin symptoms. The characteristics of the remaining 339 children are shown in Table 1. Data were missing for sex (*n* = 2), age (*n* = 3), height (*n* = 6), and body weight (*n* = 8).

### 3.2. Daily Levels of Temperature, Humidity, Atmospheric Pressure, PM_2.5_, NO_2_, Ozone, SO_2_, and AD Particles

Table 2 shows the daily levels of temperature, humidity, atmospheric pressure, wind speed, PM_2.5_, NO_2_, ozone, SO_2_, and AD particles from 1 February 2015 to 28 February 2015.

### 3.3. Skin Symptoms

Table 3 shows the odds ratios for an IQR increase in skin symptoms based on levels of PM_2.5_, NO_2_, ozone, SO_2_, and AD particles. Increases in the levels of PM_2.5_, NO_2_, ozone, SO_2_, and AD particles were not related to an increased risk of skin symptom events in children either with or without atopic dermatitis. The cumulative total summary of the symptom score was 5395 (56.8%) for a score of 1, 800 (8.4%) for 2, 84 (0.9%) for 3, and 3213 (33.8%) for non-observed. In a two-pollutant model adjusted for NO_2_, ozone, and SO_2_, PM_2.5_ and AD particles were not associated with risk of skin symptom events (Table 4 and Table 5).

## 4. Discussion

A number of studies have unmasked the deleterious effects of ambient PM on internal organs [2,3,4]. It has gradually become clear that there are four potential mechanisms by which ambient PM exerts adverse effects on the skin, including generation of free radicals, induction of cutaneous inflammatory cascades, activation of an aryl hydrocarbon receptor (AhR)-dependent mechanism, and alteration of cutaneous microflora [5,28]. However, to the best of our knowledge, the epidemiologic evidence focusing on the effects of ambient PM on skin health remains limited. Especially, in the relationship between short-term exposure to ambient PM and skin health, current evidence is lacking. Therefore, the present study investigated associations among daily PM_2.5_ and skin symptoms in schoolchildren, but found no significant relationship. Similarly, NO_2_, ozone, SO_2_, and AD particles were also not associated with skin symptoms. These results suggest that short-term exposure to air pollutants does not have an impact on skin symptoms of schoolchildren in Japan.

Kim et al. recently completed a small longitudinal study elucidating a significant association between outdoor levels of PM_2.5_ and skin symptoms in children with atopic dermatitis [29]. They also concluded that NO_2_ and volatile organic compounds may aggravate atopic dermatitis; thus, patients with skin disease may be more sensitive to exposure to PM_2.5_ than are subjects without skin disease. In the current study, our statistical analysis was adjusted for the presence of atopic dermatitis as an individual characteristic. Notwithstanding, irrespective of the presence of atopic dermatitis, the present study found no association with PM_2.5_ and skin symptoms in schoolchildren.

Kim et al. estimated the seasonal associations among skin symptoms and PM_2.5_ for winter, spring, summer, and autumn. They found a significant association with skin symptoms and PM_2.5_ only in winter, which exhibited the highest PM_2.5_ level among the four seasons. The levels of PM_2.5_ in Seoul, South Korea, where research by Kim et al. was conducted, exceeded 20 μg/m^3^ and were higher than those reported in the present study. Accordingly, the difference in results between the study of Kim et al. and the present study may depend on differences in levels of PM_2.5_. Alternatively, low-concentration or short-term exposure to PM_2.5_ may be insufficient to aggravate skin symptoms.

There are many reports suggesting that PM_2.5_ contributes to various skin diseases, such as inflammatory skin disease, androgenetic alopecia, and skin cancer [30,31,32]. Furthermore, Vierkötter et al. found a strong association of premature skin aging with exposure to PM_2.5_ in the elderly [33]. Similarly, other air pollutants, such as NO_2_, ozone, and volatile organic compounds, also have adverse effects on skin health [31,34]. Recent studies suggest the possible mechanisms by which air pollutants such as PM_2.5_ cause adverse skin effects; such mechanisms include increasing inflammatory cytokines, reducing the barrier function of skin, and activating reactive oxygen species and AhR [2,3,4,6,33]. Thus, PM_2.5_ may adversely affect skin health in schoolchildren. To better estimate the effects of PM_2.5_ on skin health and skin symptoms in children, objective evaluation methods such as score of intrinsic and extrinsic skin aging may be needed.

An important aspect of dust storms in relationship to health issues is the amount of PM_2.5_ that they contain. AD is also a serious health concern because of the associated heavy pollution, and it has been associated with increased mortality, emergency treatment for cardiovascular diseases, and hospitalization for pneumonia [14,35,36,37]. Recently, several studies from Japan demonstrated an association between AD and skin symptoms in healthy adult subjects [10,11,38,39]. Therefore, the present study also estimated the daily levels of AD particles and skin symptoms in schoolchildren using LIDAR data but found no such relationship. However, the studies reporting a significant association with AD and skin symptoms estimated the effect of heavy AD on skin health. In these studies, heavy AD was defined as a density greater than 0.6 km^−1^ or 1.0 km^−1^. In the present study, the average level of AD particles was 0.02 km^−1^. Thus, low-concentration exposure to AD particles may be insufficient to aggravate skin symptoms. Alternatively, the effects of AD on skin health may differ between adults and children.

There are several limitations in the current study. First, the study duration, which was one month, may not have been long enough to estimate the effects of short-term exposure to ambient PM on skin health. However, our previous one-month study found a significant negative association with ambient PM and respiratory function in schoolchildren [23], suggesting that one month may be sufficient time. Second, this also restricted any assessment of seasonal variation. Regardless, according to the results of Kim et al. [29], PM_2.5_ during winter was the most concerning with respect to the deleterious effects of ambient PM on skin health. Thus, although the present study was conducted during the most suitable season, such effects may depend on the composition of PM_2.5_. Third, skin symptom scores were not validated because validated skin symptom scores were not available in Japan at the time of the study. Therefore, as in previous studies which estimated the association with PM_2.5_ and respiratory symptoms, skin symptom scores were reported as 0 (no symptoms), 1 (mild symptoms), or 2 (severe symptoms) [40]. Finally, we were unable to measure the individual amount of exposure to particulate air pollutants.

## 5. Conclusions

Short-term exposure to PM_2.5_ was not associated with skin symptoms in schoolchildren. AD particles are an important source of PM_2.5_ in Japan. However, there was no relationship between AD particles and skin symptoms. Neither PM_2.5_ nor AD particles aggravated the skin symptoms of schoolchildren.

## Figures and Tables

**Table 1:** Characteristics of the 339 children included in this study.

Characterristics	Value	
Boy/Girl (number)	170/167	
Age (number)		
10-year old	42	(12.4)
11-year old	293	(86.4)
12-year old	1	(0.3)
Height (cm)	144.6	±7.1
Weight (kg)	36.4	±7.1
Allergic disease (number)		
Asthma	36	(10.6)
Allergic rhinitis	54	(15.9)
Allergic conjunctivitis	8	(2.4)
Atopic dermatitis	26	(7.7)
Food allergies	16	(4.7)

Data are shown as mean ± standard deviation or *n* (%).

**Table 2:** Average meteorological and air contaminant levels from 1 to 28 February 2015.

Valuables	Value
Temperature (Celsius)	5.4 ± 2.3
Humidity (%)	72.1 ± 8.0
Atmospheric pressure (hPa)	1016.6 ± 5.5
Wind (m/s)	4.0 ± 2.0
PM_2.5_ (μg/m^3^)	13.0 ± 7.5
NO_2_ (ppb)	2.5 ± 1.5
Ozone (ppb)	38.1 ± 7.0
SO_2_ (ppb)	3.0 ± 1.9
Asian dust particles (km^−1^)	0.02 ± 0.03

Data are shown as mean ± standard deviation.

**Table 3:** Multivariate analysis using generalized estimating equation (GEE) logistic regression models to assess the association between skin symptoms and interquartile range (IQR) changes in the air contaminant concentrations.

Exposure Metric	Children (*n* = 339)			
	IQR	Odds Ratio	95% CI	*p* Value
PM_2.5_	10.1 μg/m^3^	1.03	0.80 to 1.33	NS
NO_2_	1.9 ppb	0.99	0.80 to 1.23	NS
Ozone	11.3 ppb	0.79	0.56 to 1.12	NS
SO_2_	2.5 ppb	1.07	0.85 to1.35	NS
AD particles	0.01 km^−1^	0.99	0.92 to 1.06	NS

CI, confidence interval; NS, not significant.

**Table 4:** Estimated effects of PM_2.5_ on skin symptoms in the two-pollutant model after adjustment for NO_2_, ozone, and SO_2_.

Adjustment	Odds Ratio	95% CI	*p* Value
Adjusted for NO_2_	1.20	0.65 to 2.24	NS
Adjusted for ozone	0.98	0.72 to 1.34	NS
Adjusted for SO_2_	1.05	0.83 to 1.33	NS

CI, confidence interval; NS, not significant.

**Table 5:** Estimated effects of AD particles on skin symptoms in the two-pollutant model after adjustment for NO_2_, ozone, and SO_2_.

Adjustment	Odds Ratio	95% CI	*p* Value
Adjusted for NO_2_	0.98	0.89 to 1.08	NS
Adjusted for ozone	0.95	0.88 to 1.03	NS
Adjusted for SO_2_	0.99	0.92 to 1.06	NS

CI, confidence interval; NS, not significant.
